# The body in language, the language beyond the body: embrainment and graded embodiment in the evolution and use of language

**DOI:** 10.3389/fpsyg.2026.1774505

**Published:** 2026-04-10

**Authors:** Michael A. Arbib, Valentina Cuccio

**Affiliations:** 1 Computer Science and Neuroscience, University of Southern California, Los Angeles, CA, United States; 2 Department of Ancient and Modern Civilizations, Università Degli Studi di Messina, Messina, Italy

**Keywords:** cultural evolution, embodied simulation, embrainment, graded embodiment, large language models, metaphor, second nature, tower of abstraction

## Abstract

We reject the notion that the assertion “language is embodied” has a yes/no answer and instead introduce an account of a *gradient of embodiment* that ranges from “strict embodiment” restricted to elements of language describing humans engaged in practical bodily interaction to fully abstract concepts. We show how concepts may blend strict embodiment and abstraction, and demonstrate that strictly embodied knowledge may ground linguistic meaning phylogenetically and ontogenetically. Much adult language use resides “high” in a *tower of abstraction.* However, movement away from strict embodiment need not imply movement only up the tower of abstraction. Embodied simulation combined with metaphor provides one mechanism for linking abstraction to embodiment. An account of biocultural evolution shows how changes primarily in brain structure (*embrainment*) made possible the linkage of practical action to pantomime in grounding protolanguages and how cultural evolution built on this to create a symbolic and physical environment that depends on the availability of grammar to create metaphors and new abstractions. Critiquing the claim that large language models mirror the human brain’s language system we show how human embrainment links disembodied processing to processes that are strictly embodied.

## 1 Introduction

### 1.1 Strict embodiment, abstraction, and embrainment

We will only consider languages in the sense of human languages with rich lexicons and grammar, such as Armenian, Swahili, and Chinese. Certainly, such languages and signed languages arose as something used by humans with bodies in social interaction, but with the invention of writing for spoken languages, one could point to words written on the page and say “that is language” and so, perhaps, they are not embodied. To this one might reply that language can only, almost literally, leap from the page when it is read by an embodied human.

The central question for this paper is “What do we mean by “the body” in embodiment when we discuss embodied cognition or language?” For many writers, *embodiment* refers to the whole person, integrating the physical body with the cultural and social context. This opposed the view that language or cognition could be understood solely as neural processes. We agree that body and brain form an integrated system fundamentally shaped by the social and cultural environment in which each individual grows and to which they may contribute. However, to assert that “human language results from the interaction of brain, body, and environment” risks falling into tautology. We introduce the notion that the use of language is *strictly embodied* to the extent that it engages the sensory and motor activity of a human body engaged in practical activities. We thus deny that *all* language use is *strictly embodied* (setting aside those sensorimotor activities explicitly involved in human production and perception of language).

While apes share much of their bodily structure with humans (despite important differences, such as the transition from fur to hair, the emergence of bipedalism, the evolution of menopause, and the dexterity supported by opposable thumbs), it is plausible to argue that *embrainment*, properties attributable to the brain rather than the form of the rest of the body, enabled the cultural evolution that underpins modern human cognitive and linguistic abilities (Northoff, 2021).

We build on the notion of *second nature* in the sense of Gehlen (1940), who wrote (as translated from the German) “Our nature is largely second nature, and our second nature is the way it is not just because of the potentialities we were born with, but also because of our upbringing, our development,” and of McDowell (1976). Cuccio (2024) argued that “Rationality and culture, in this perspective, would emerge from our biology (our first nature), from which we develop species-specific cognitive capacities.” Our embrainment was essential in complementing basic capabilities of the body in underwriting the happenstance patterns of cultural evolution that enabled us “to communicate through symbols and, therefore, to reason, to produce culture, and to learn through socio-cultural processes. Logical-linguistic thought, reason, and culture have enabled us to develop new technologies whose impact on the biosphere has been disruptive.”

Culturally grounded abstract concepts arise from collective practices that accumulate over generations, becoming part of a shared symbolic environment. They are cultural products through and through, yet they become part of second nature insofar as individuals learn to navigate these conceptual systems effortlessly. Human beings are distinctive in their ability to stabilize and transmit such constructs cumulatively, a dynamic sometimes described as the “ratchet effect” (Tennie et al., 2009). By contrast, the abstract concepts produced through hierarchical categorization depend on a different manifestation of second nature: our evolved capacity to generalize from experience to group entities into classes, and to construct increasingly inclusive or more focused categories. Animals, too, generalize across encounters, for example, by learning to identify novel edible items and implicitly forming functional subcategories that guide foraging, but we argue that our extended capacity to construct abstract categories was phylogenetically enabled by our *embrainment*, structural changes in neural architecture that enhanced the possibility for cultural evolution to support new forms of conceptual processing.

A *Homo sapiens* child would have developed a recognizably human body and a brain with the same *potential* for development—but that brain’s *development* would be drastically different because the physical and symbolic environment has changed so much in the last 200,000 years. As explored in §3. we say that this ancient human brain was already *language-ready* in that it could support the cultural evolution that led modern humans to be *language-using*, with symbolic communication supporting much of reasoning, cultural learning, and abstract thinking—the use language, supporting as it does both strict embodiment and abstraction, is made possible for us among all the great apes because of our distinctive brain architecture and its ability to be shaped by the current physical and social environment. *Humans* develop in a physical and social environment that was shaped by human communities long before they were born and so must be introduced to them through social learning practices. Developing and living within this enculturated ecological niche profoundly transform our first nature, our body and brain, thereby contributing to the varied forms of second nature make us human. More specifically, much of language is not related directly to the specifics of having human bodies and basic sensorimotor and emotional capacities. In subsequent sections we introduce the notion of the “tower of abstraction” (Arbib et al., 2014) that supports (but is not coextensive with) a “gradient of embodiment” that ranges from “strictly embodied” language to “disembodied” (≈ highly abstract) language. However, with (Arbib et al., 2014), we still see cultural evolution of languages and the child’s acquisition of language as building on (but not limited to) strict embodiment. Our thesis, then, is that *some* language use may be either strictly embodied or based on the recognition of behavior in other humans that is strictly embodied for them.

### 1.2 Interlude

Language goes far beyond words labeling concepts. It provides the ability to employ grammar to create phrases and sentences to capture novel meanings, and some of these may create (not just label) new concepts. When we use a word or phrase, then, the use may be strictly embodied, but on other occasions, the meaning may depend on our ability to repurpose a word by using metaphor to move it into a new domain, or by using language to help us attend to further aspects of strictly embodied experience or by supporting processes of abstraction. Some examples like “milk” or “grasp” have strictly embodied meanings for most people that can then be extended by metaphor, others like “justice” or “philosophy” are high on the tower of abstraction. We consider “milk,” “dial” and “justice” to provide the reader with some intuitions as background for the sometimes embodied but often more abstract use of language in the following sections.

#### 1.2.1 Milk

The primal experience of milk for many humans is the strictly embodied experience of suckling at the mother‘s breast but few humans can recall this experience by the time they are acquiring their first association with the word “milk” drunk from a bottle or a glass with a sense of its color and its taste and feel in the mouth, but with no association with the origin of the fluid.

In the rural past, most children would have later seen a cow or other animal being milked and two different meanings, “what I drink” and “what comes out of the animal’s udders,” would coalesce into one more complex meaning. This already introduces a distinction between those with the strictly embodied experience of milking a cow and those who have only observed someone else doing the milking. This is an important distinction to which we return in our discussion of mirror systems in §2. However for most children today in industrialized countries, “milk” is the fluid that comes from a distinctive type of cardboard box. The association with its source from female mammals may be learned much later, with its understanding being part of acquiring (by use of language other than metaphor) an understanding of the abstract concept “mammal,” even to the point of accepting the apparently ridiculous statement that “A whale is not a fish.” However, the word “milk” can be used metaphorically as in “the milk of human kindness,” an abstraction which may be grounded in the mother’s giving of milk to the child and so might be strictly embodied only for fond mothers. However, we have here a meaning of “milk” that is generally licensed only by this particular context or others that evoke it. These usages exemplify different degrees of embodiment.

#### 1.2.2 Dial

Being active in the day and sleeping at night (we evolved as diurnal animals) is part of our strict embodiment though observing the daily motion of the sun and the nightly motion of the stars (in our ground-centered frame) no longer depends on the form of our bodies. The invention of the *sundial* involved two very powerful abstractions: the ability to count and the idea that time could be measured. With this consider the chain of metaphors for *dial*, from sundial to clock dial to rotary telephone dial to pushing numbers on a smartphone. Ironically, we pass through abstract ideas to end up with a new strictly embodied function, but one engaging the abstraction of the digits (bodily metaphor) from 0 through 9 in a manner far removed from counting. What is noteworthy is that this complex technological and etymological history plays no role in understanding the present-sense of the word *dial*—by a long chain of metaphors it has returned to a strictly embodied action made possible by the technology of smartphones and the communication network that they exploit. Note the important point that, especially in light of changes in the environment, while metaphors may be a tool for moving up the tower of abstraction, they need not be, and that processes of abstraction may anchor new forms of strictly embodied language.

#### 1.2.3 Counting

Counting on one’s fingers from 1 to 10 may, for some people, be an embodied correlate of the notion of numbers, but Fischer et al. (2012) introduce a collection of research papers that make clear that there is no agreement about the relevance of finger counting for numerical cognition. The Babylonian number system used base 60 (the source of our 60 min in the hour). While Roman numerals use base X, the expression of very large numbers became cumbersome. It was only the invention of the “no finger” numeral 0 by Brahmagupta around 650 CE that made possible the power of Arabic notation (invented in India), a power increased by the decimal point. In the telephone dialing example, strings of numerals are names, not counting numbers. Finally, even if the numbers 1 through 10 were strictly embodied, the number 23,804,353 only exists at a high level of abstraction that expresses a second nature made possible by our embrainment rather than any change in bodily form.

#### 1.2.4 Justice

When their mother cuts a piece of cake for her two children, the aggrieved child with the smaller piece may howl, “That’s not fair.” But this early (and limited) experience of *fairness* is a long way from understanding the highly abstract notion of *justice* which is high up the tower of abstraction, and it takes verbal analysis of a lot of examples of behavior to get you to the point where you can talk about justice. We must go beyond metaphor to the power of language to employ grammar to construct narratives that give us the power to introduce new words with rich conceptual associations. But when we have that, we can come “down the tower of abstraction” to say that a specific example of courtroom behavior was an example of justice or a case of injustice.

### 1.3 The gradient of embodiment

The notion of *graded embodiment* refers to a gradation that starts with concepts and linguistic meanings that depend on direct *bodily knowledge* and extends to more and more abstract concepts made possible only through the *cultural evolution* through which our conceptual systems and languages emerged from their more embodied precursors (see §3).

We reiterate that the term *body* here refers specifically to the human body, and that *action* designates those actions human speakers are capable of performing. This can get fairly subtle. By this criterion, “Birds are flying” is not strictly embodied because it is based on observations that do not depend on the body of the observer. Some actions are innate, such as swallowing. Others are profoundly shaped by cultural experience, such as dancing. The interplay between nature and nurture that characterizes the notion of second nature becomes especially apparent in actions involving tools. Take the action of “writing”: it is possible only because we possess a highly enculturated brain that has acquired language and mastered the symbolic skill of producing marks on paper or on a screen to represent linguistic units. At the same time, writing depends on, and is constrained by, our sensorimotor abilities. Its execution therefore requires both culturally acquired knowledge and bodily capacities. As in the dial example, many embodied concepts resulted from our embrainment that could support our mastery of a culturally evolved environment.

Consequently, concepts and meanings can be positioned along a continuum of embodiment, ranging from highly embodied notions, such as action-related meanings (e.g., the verb “to grasp”), to concepts such as “justice” that do not depend directly on sensorimotor experience for their formation but may be abstracted from diverse situations or narratives, some that do and others that do not exemplify the concept (as in defining *justice* in part by understanding *injustice*). Our symbolic abilities provide an essential, though culturally modulated, dimension of our embrained cognition, enabling us to construct meanings that transcend immediate physical experience. At the same time, the cultural environment in which we are embedded provides the material objects, institutional structures, and patterned behaviors that serve as affordances and interpretive frameworks, guiding how such concepts are learned, negotiated, and stabilized. Through these intertwined cognitive and cultural processes, humans are able to develop abstract concepts that remain largely, and sometimes entirely, disembodied, playing a central role in our intellectual, moral, and social lives, along with the embodied ones.

As our examples in §1.2 show, it is hard to adjudicate that one concept is strictly embodied and another is not. The concept of *milk* as we drink it is strictly embodied, but the concept of *milk* as “a white fluid in a carton with the word “milk” on it” depends on our culturally evolved embrainment rather than on any particularities of our bodily form. In this spirit, we might want to refer to concepts as being *concrete* rather than strictly embodied if they are not directly rooted in our own motor system but are low on the gradient of embodiment, being rooted in our systems for perception of objects related to basic actions (while noting that the notion of “basic” is itself graded). For example, the concepts of a *river* and its *bank* do not depend on human bodily form, yet their perception is very much part of our second nature. Consider the distance from drinking and tasting water and reducing our thirst (an embodied characterization we may share with many nonhuman creatures but not fish) to recognizing all the qualities that distinguish a river from a handful of water we can drink. Moreover, our example of “dial” showed that as a result of human cultural and material evolution, even direct sensorimotor experiences can only make sense if embedded within a wider contextual frame than was available to our distant ancestors. In particular, the ability to count from 0 to 9 and name each number bears no relation to the basics of the human body. All this reinforces the importance of gradation rather than binary distinctions. The general issue of how strict embodiment varies for humans with different bodily forms remains open, but (outside our scope) the impact of being male or female can certainly affect what is strictly embodied for each of us. The view of Embodied Simulation espoused in §4 goes beyond the motor system to also include perception areas and shows how bodily metaphors may change the conceptual reach of words. When we say “She grasped the idea” it is clear that metaphor has moved “grasp” from our embodiment to a more abstract realm. Moreover, our description of a possible relation between these concepts may involve a bodily metaphor, as when we say, “The river swallowed the bank.” The issue can become complex. For example, one may recognize objects as being books even if one has no concept of reading—but one does not understand the concept *book* unless one understands its relation to reading. This suggests that “turning the pages of a book” is a body-based action whereas “reading a book” takes us via cultural evolution far up the gradient of embodiment to a behavior that depends crucially on aspects of embrainment that take us beyond the basic sensorimotor repertoire of practical action.

Some concepts and meanings emerge entirely from cultural practices. Consider, for instance, the domain of bureaucracy: its concepts refer to roles, procedures, and institutional structures that exist only within symbolic and cultural systems. They are unintelligible outside a sociocultural context. Yet, despite their fully cultural and symbolic origin, we often understand and describe these concepts in bodily terms. Bureaucratic hierarchies, for example, are conceptualized using schemas of verticality, control, and physical elevation, someone “at the top,” a subordinate “below,” an authority “overseeing” others. In these cases, we metaphorically re-anchor a culturally constructed notion in bodily experience. In these cases, we project embodied schemas onto concepts that are, in essence, products of culture rather than of sensory or motor engagement with the world.

It is crucial to note that emotions and drives involve visceral sensations and physiological states. “Basic” emotions like *rage* are strictly embodied, but language also gives us terms for “less embodied” aesthetic emotions, or enculturated emotions like *Schadenfreude.* Similarly, certain interpersonal relations such as fighting, fleeing, and caregiving are part of our basic embodied repertoire. Combining these two concepts, the facial expression of emotions seems to be another basic part of our embodiment crucial for social interaction.

### 1.4 The tower of abstraction ≠ the gradient of embodiment

The notion of a “tower of abstraction” can be understood in two complementary ways, each highlighting a different dimension of how concepts are formed and organized:

In the first sense, the tower refers to a continuum that stretches from strictly embodied (and concrete) to fully abstract concepts. Some meanings remain deeply anchored in sensorimotor experience, others draw only partly on bodily schemas, and still others, typically those shaped by cultural practices, may be largely or wholly disembodied. Language lets us define new concepts by explicit definition and by appealing to what may be a whole system of narratives. *Mammal* can be formally defined counter-intuitively as a “a creature of a species in which mothers suckle their young” whereas notions of *God* or *gods* may evolve from the myths of different religions, while *justice* may emerge through diverse narratives to gain meanings that can then be applied in embodied situations, but in ways that vary from culture to culture and may vary from person to person. A concept’s location on this continuum reflects the extent to which bodily experience contributes to its comprehension.

The second sense of the tower concerns our capacity to construct conceptual hierarchies. Abstraction may proceed by moving from a category such as *apple* to more general or more specific categories, such as *fruit* or *red apple*, respectively.

From these two senses emerge two principal routes by which abstraction develops. One route involves the cultural elaboration of meanings that already sit high on the embodied–abstract continuum, as in philosophy, mathematics, or law. Such domains give rise to concepts such as *differential equation* and *justice* whose content depends not on bodily experience but on historically accumulated symbolic practices. The other route consists in the formation of category hierarchies through processes of generalization and specialization. More generally, both mechanisms may work together in forming new concepts.

Though these pathways may lead to different kinds of abstraction, both ultimately rely on what we call our “second nature.”

We are transferring the search for embodiment from its personal fluctuations during use to cultural evolution and individual language acquisition. The issue for graded embodiment is the number of steps, whether through chains of metaphor or through narratives or through experience in the use of words in the company of other words that are required to understand the concepts. Strikingly, the “degree of embodiment” may be high when we consider the etymology of a word, and yet low when we chart the path required by an individual person to acquire a *current* socially accepted meaning (as in the *dial* example). Indeed, we often use a word without knowing its etymology or recalling how earlier generations used it. At one stage of English, “awful” meant “full of awe; awe-inspiring.” Now we learn it as a word for denigrating something without pausing to think about the first syllable.

### 1.5 How we proceed

Having made explicit the notion of graded embodiment and related it to the tower of abstraction, we can now chart the course we follow in the remaining sections.

Unfortunately, the notion of mirror neurons or systems was over-interpreted in the popular press and elsewhere, leading (Hickok, 2014) to declare that was a “myth of mirror neurons,” and (Arbib, 2015) to show that the “myth” could be dispelled by attending to the primary literature, and stressing how much of “mythical” functionality was in fact supported by consideration of how mirror neurons act with brain systems “beyond the mirror.” §2 thus not only introduces the notions of mirror neurons and mirror systems but offers a small sample of evidence for the need to consider those other regions of the brain.

The next two sections offer two approaches to the embodiment of language in which mirror systems play a crucial role. In some ways these two accounts have been seen as opposed. A major concern of this paper is to re-assess them within the broader context of understanding graded embodiment and the tower of abstraction. §3 summarizes the Mirror System Hypothesis for the evolution of the language-ready brain but renames it as the Beyond the Mirror Hypothesis to stress the changes beyond the mirror that distinguish the embrainment of our last common ancestor with the great apes (LCA-ga) from the embrainment of *Homo sapiens.* It charts an evolutionary path from gestural communication to pantomime and then protolanguage as a mechanism for social coordination and teaching, and then sketches how cultural evolution could have led to grammar and narrative. §4 then shows how embodied simulation and bodily metaphors may begin to move us beyond strictly embodied words and concepts, while stressing that mechanisms beyond the mirror must be involved to construct different domains of meaning and support the way in which metaphor can operate to transform the meaning of a word in a source domain to develop a meaning for the word in the target domain.

§5 then changes course to consider a different line of attack on the claim that language is embodied. This attack is rooted in the development of Large Language Models (LLMs) and their amazingly proficient deployment of language to respond to natural language prompts. While reflecting on the neurological syndromes of apraxia and aphasia, we reject the claim that LLMs model the language system of the human brain, but nonetheless find it useful to assess what this AI “disembodiment” means for the claim that human language use is grounded in strict embodiment through evolution and in the child’s development and yet the human brain supports the tower of abstraction.

Finally, in §6, we bring these various strands together to assess what is offered by the gradient of embodiment and the tower of abstraction, and offer some implications for future research.

## 2 Mirror neurons, mirror systems, and systems beyond the mirror

Mirror neurons in the macaque and mirror systems in humans contribute to recognition of actions in the agent’s repertoire but other brain regions must contribute to the recognition of other actions and to imitating actions not yet in the agent’s repertoire—thus the slogan that mirror systems interact with brain systems “beyond the mirror.” §§3 and 4 offer approaches to the graded embodiment of language whose embrainment involves, but in general will go beyond, mirror systems.

### 2.1 From mirror neurons to mirror systems

Using single-cell recording of neuron firing to study hand-related neurons in an area called F5 in monkey premotor cortex, di Pellegrino et al. (1992) found that some neurons were active not only when the monkey carries out certain manual actions but also when the monkey observes an other (monkey or human) carrying out a similar action. They were subsequently called *mirror neurons* (Gallese et al., 1996). However, the neurons active when the monkey performs an action are *not* the same as those active when the monkey observes a similar action. The discovery of mirror neurons shows only that these two populations overlap.

Some neurons in area F5 are responsive not only for visual observation of actions associated with characteristic noises (such as breaking a peanut or tearing paper) but also for the sounds of these actions (Kohler et al., 2002). Since the monkey brain did not evolve to tear paper, it is clear that mirror neurons for specific types of action are not genetically specified—rather, the genes specify learning mechanisms that support the emergence of mirror neurons (Oztop and Arbib, 2002). In addition, there are *oro-facial mirror neurons* (located in an area adjacent to the manual action related neurons) that become active during the execution and observation of mouth actions related to *ingestive* functions. Further, the hand and mouth mirror networks partially overlap to support hand-mouth synergies for praxic actions like eating and drinking but also for communicative purposes in order to better convey and control social signals. Additionally, some oro-facial mirror neurons may have *communicative* mouth gestures as their most effective visual stimuli. Ferrari et al. (2017) show that, unlike the F5 neurons for manual actions, the oro-facial motor neurons are connected with limbic structures involved in emotions and reward processing. This reminds us that emotions provide a crucial form of embodiment but—like practical actions and the environment they act with and upon—they are transformed by cultural evolution.

Using brain imaging rather than recording from single neurons, researchers studying the human brain have found various mirror systems—a *mirror system for a class X of actions* is an area more active, against a baseline set by control tasks, both when the subject performs an action from X and when the subject observes another performing an action from X. The *human* brain has a mirror system for manual actions (Grafton et al., 1996; Rizzolatti et al., 1996), another mirror system in a very different area of the brain is related to disgust (Wicker et al., 2003), and so on.

### 2.2 Beyond the mirror

Gazzola et al. (2006) used fMRI to search for human brain areas that respond both during motor execution and when individuals listened to the sound of any action made using the same effector. They found two such systems: one more involved during listening and execution of hand actions, and another more involved for mouth actions. They then asked: “Would people with stronger mirror system activity for these two classes of actions tend to show more empathy for others?” To test this, they employed a questionnaire for assessing how empathic each subject was and, indeed, found (statistically) greater activation of the mirror systems in those individuals who scored higher on the empathy scale. This suggests a link between the mirror systems and empathy—but only a partial link. Differences in mirror system activity explained only around 40% of the variance on the empathy test. This suggests that people who are more observant of others’ (visible and audible) actions may be somewhat better at inferring their mental states. This finding reinforces the finding that we must not talk of *one* mirror system in isolation, but rather seek to understand the roles of, and interactions, between, multiple mirror systems and systems “beyond the mirror.”

Mirror neurons offer a key contribution to social neuroscience. If one can recognize what someone else is doing or their emotional expression and relate it to one’s own, then that recognition may be crucial to guiding your interaction with the other. However, we reiterate that one’s behavior may also depend more importantly on recognition of actions outside one’s own repertoire (as in watching birds fly). Significantly for neural reuse, one may use that recognition and understanding of the goal the other seeks to achieve to enrich your own capability to learn how to *imitate* that action and the contexts in which to deploy it. But if you are learning a novel action then there cannot be mirror neurons for it—you can recognize it before you can have neurons that fire both for recognition and execution. Moreover, having mirror neurons alone does not guarantee the ability to imitate. *Monkeys do not imitate to any great extent, and so mirror neurons by themselves do not support imitation*. Yet apes have simple imitation and we have complex imitation (§3), implying that evolving embrainment integrates mirror systems with other neural systems *beyond the mirror* that are not present in our closest relatives in bodily form, the great apes.

In another study, this time with soccer players, Tomeo et al. (2012) found that the goalkeeper had a very different response to videos from that of other players. This suggests that action recognition (whether or not it involves mirror neurons) may be implicated in “what one does next” perhaps even more than in emulating the observed action. Newman-Norlund et al. (2007) assessed mirror system activity in participants, finding that BOLD signal in the right inferior frontal gyrus and bilateral inferior parietal lobes was greater during preparation of complementary than during imitative actions.

Buccino et al. (2004) considered the mirror system for oro-facial movements. fMRI was used to scan the brains of humans in six conditions. In three, the subject saw videos (without sound) of a person taking a bite of food, a monkey taking a bite of food, or a dog taking a bite of food. They found that in each case the subject can map the act of biting onto their own actions to activate a mirror system *for biting*. In the other three, the subject sees a video (again, no sound) of a person talking, a monkey making communicative oro-facial movements like teeth chattering and lip smacking, and a dog barking. The fMRI shows vigorous mirror system activity when the subject views the human, only modest activity when viewing the monkey and none when viewing the dog.

This reinforces the point that *we don’t need mirror neurons to recognize actions, but that mirror neurons can enrich perception of actions when they fall within our own* repertoire. As in watching a bird fly, “concrete” actions cannot engage mirror neurons for flying like a bird does but may engage other forms of both perceptual and motor “simulation” that are included in the account of Embodied Simulation in § 4.

## 3 Beyond the mirror to language

In this section, we explore the Beyond the Mirror System Hypothesis (BtMH) on how biocultural evolution endowed our ancient ancestors with a *language-ready brain* (§3.1) and how cultural evolution then reshaped our brains and environments so that languages can exploit grammar and narrative in undergirding our second nature (§3.2). §4 will then explore *embodied simulation* and show how bodily metaphors provide one important basis for moving language a step up the tower of abstraction while expanding grammar and narrative support further enrich language.

### 3.1 The early stages

The Mirror System Hypothesis (MSH)^1^ was so named because of two observations (Arbib and Rizzolatti, 1997; Rizzolatti and Arbib, 1998): (i) The mirror neurons for manual actions discovered in macaque were in a brain region homologous to human Broca’s area; and (ii) although Broca’s area was traditionally linked to speech production, damage there could also yield aphasias of sign language production (Poizner et al., 1987). Together, these supported the notion that manual skill could provide a scaffolding for the evolution of language with the *parity* property: that the meaning of utterances produced by a “speaker” (or signer) could be (more or less) recognized by other members of the same language community.

However, *mirror neurons play little role in the theory*. Thus, despite decades of the MSH label, we now change the name of the theory to *the Beyond the Mirror Hypothesis* (BtMH) to emphasize our concern with the dramatic changes in embrainment that have been required to enable humans to evolve and use language. This section charts a path for biocultural evolution from our last common ancestor with the great apes (LCA-ga) involving increasing embrainment for bodily actions like action recognition, imitation and pantomime to yield a “language-ready brain.” It then took much cultural evolution to develop “language-using brains” that could use language to mix embodied concepts with concepts far up the tower of abstraction.

BtMH posits that, like today’s great apes, LCA-ga had a capacity for “simple” imitation in recognizing subgoals (including the availability of affordances) but—if the action is not recognized as one already in its repertoire—the animal uses trial-and-error to develop actions to pass from one subgoal to the next rather than paying attention to trajectories whereby those subgoals were reached (Byrne and Russon, 1998). The transition from LCA-ga to *Homo sapiens* (and possibly Neanderthals) involved *biocultural* evolution (changes in genome for brain and body as well as changes in social practice to exploit the new potentialities) that yielded capacities for complex action recognition and imitation and for pantomime that in turn support the emergence of an expanding spiral of protolanguages:

#### 3.1.1 Complex action recognition and imitation

This combines *complex action recognition*—the capacity to “parse” another’s behavior into constituent actions, recognizing familiar actions and also attending to unfamiliar actions, but adding attention to the motion as well as the goals of subactions—with *complex imitation*, which exploits this ability to, possibly, achieve a first approximation to that action *without* trial and error.

At this stage we are still focused on “strongly embodied” actions, but adding imitation which in some sense reverses the mirror property—bringing into one’s own repertoire an action that one comes to recognize an other performing to the benefit of themselves or others.

But how do we get from embodied actions to words that describe those actions or the agents or objects involved in that performance?

BtMH posits that, whatever the merits of vocal signaling, the crucial innovation was based on pantomime. Russon (2018) demonstrated that great apes exhibit some “pantomime-like” behavior. However, BtMH posits that this capability gained complexity as part of the long path of biocultural evolution in the 5–7 million years that separate us from LCA-ga. Pantomime involves a crucial change from *transitive* actions (actions upon objects, guided by the object’s affordances) to *intransitive* actions (conducted in the absence of objects). This transition is important in teaching new skills (Gärdenfors, 2021, 2024). Briefly—see Arbib (2024) and Chapter 8 of Arbib (2012) for details, the notion is that protohumans first discovered the ability to perform truncated and intransitive forms of an action to communicate “in the present context, there is something about this action that is relevant to what I am trying to communicate” (long before such a sentence was thinkable let alone expressible in language!). We call this ability the *ad hoc* pantomime system, the ability to freely pantomime performances with the expectation that the observer will interpret this correctly as a request or instruction (We distinguish the early phylogenetic development of *ad hoc* pantomime from present-day conventionalized pantomime).

This embrainment involves (i) the emergence of two distinct neural representations—extending that for performance of actions to support ad hoc pantomiming of actions and (ii) the social capacity for one creature to generate a pantomime to request an other to perform in a certain way, and the ability of the other to recognize the pantomime as a communicative act rather than a failed practical act.

For creatures who reach this stage, communication by *ad hoc* pantomime becomes part of their second nature. But *ad hoc* pantomime is not enough to support language. Two steps are involved. One is that *ad hoc* pantomimes may become ritualized—a group may come to use a simplified form of the pantomime that requires less effort to produce and recognize. But this then opens the way for conventions to arise that modify the original to make important distinctions. For example ac pantomime for “bird flying” can be changed in different ways to yield signs for “bird” and “flying” (Supalla and Newport, 1978). We call the result a protosign (these are simply called “signs” when talking of modern signed languages, full human languages emerging long after the first protosigns):^2^

Protosign is a manual-based communication system whose conventionalized forms yield an open repertoire supporting parity. It is based in part on the conventionalization of pantomimes. The signs may but need not be iconic—novel protosigns may be needed to clarify pantomimic sequences as in the bird flying example or to represent concepts like “blue” for which iconic gestures are not available.

Both ambiguity in pantomime and a desire for more efficient signaling provided the “incentive” for coming up with “protosigns” as conventionalized forms with restricted ranges of meaning in certain contexts. In this way, groups acquired the understanding that new symbols can provide non-iconic messages, though the cost is that different communities may be unable to recognize each other’s protosigns. BtMH then posits that the ability to develop novel vocalizations with conventional meanings, protospeech rested on the “invasion” of the vocal apparatus by collaterals from the communication system that includes the expanded F5/Broca’s area.

The idea that protosign led protospeech has been subject to a number of critiques (see, e.g., Aboitiz, 2013; Fogassi et al., 2013) and a response (Arbib, 2013).^3^ However, our doctrine of expanding spirals specifies that when we consider two capabilities of modern humans, the development of each one may have assisted in the emergence of the other. As A becomes more complex, the result supports changes in B; as B changes this supports further changes in A; and the cycle continues to enrich the complexity of A and B. BtMH first applied this doctrine to the co-evolution of protosign and protospeech (Arbib, 2005).

Whatever the sequence of embrainments, modern human brains in language-using societies have three related and coupled neural systems for sensorimotor integration for producing and recognizing actions

1. one for practical actions,
2. one for visuo-manual communication, and
3. one for audio-vocal communication.

BtMH claims that the above “stages” together yield *early humans with language-ready brains*, and the ability to employ simple protolanguages with parity, where a *protolanguage* is a form of communication based on gestured or vocalized “protowords” (early instances of protosign and protospeech) but with a limited lexicon and little or no grammar.

To conclude this subsection, we stress that BtMH does not claim that “language is performed by mirror neurons” or that “the neural circuits that underlie, e.g., manual action in humans are identical with those that support human language use.” Rather, we have charted a series of new capabilities that distinguish humans from great apes and thus, presumably, from LCA-ga. These capabilities, we assert, require major changes in embrainment. There are no fossil brains (and very few fossils) for possible examples of LCA-ga, but there are extensive studies in comparative neuroanatomy that attests to the dramatic changes and increased complexity that distinguish the brains of modern humans from those of other primates that appear relevant to the human capacity for language (the relevant literature is outside the scope of this paper, but here are three examples: Hecht et al., 2013; Rilling, 2014; Rilling et al., 2008). It is for this reason we have suggested that the Mirror System Hypothesis might be more informatively named the Beyond the Mirror Hypothesis.

### 3.2 The emergence of grammar

With this we turn to the cultural evolution that BtMH posits led from protolanguages to languages with no more primary genome-based changes in brain and body (though secondary changes may have been relevant) but major changes in the varied second natures of enculturated communities. The critical property of the adaptive human brain for such cultural evolution is known as neural re-use or cortical recycling (Anderson, 2010; Anderson and Finlay, 2014; Dehaene and Cohen, 2007). For example, part of the brain that apparently evolved to support face recognition gets recycled in literate humans to support visual word-form recognition (Dehaene et al., 2010). Here we sketch how the human language-ready brain supported the cultural evolution of pantomime, protolanguages and languages so that development in a language-using community will reshape the brains of most children to provide circuitry that supports the production and understanding of one or more specific languages.

Our approach shares its general framework with *construction grammar* to hold that languages have a multitude of diverse constructions that combine form and meaning, rather than the approach of *generative linguistics* that advocates a syntax that operates only on forms devoid of semantics. To clarify the distinction, briefly and thus simplistically:^4^

1. *Generative linguistics* (Chomsky, 1981, 1992; Radford, 2004) posits that language production involves separate processes for syntax and semantics. Creating syntactic forms does not involve meaning. To do this it introduces syntactic categories (such as word classes like Determiner, Adjective, Noun, Preposition and other classes like Noun Phrase, Verb Phrase, or Sentence) and rules for forming Sentences hierarchically: a Sentence might combine a Determiner Phrase and a Verb Phrase, a Determiner Phrase might combine a Determiner and a Noun Phrase, and a Noun Phrase might be either a Noun or an Adjective followed by an already defined Noun Phrase, with variations upon the syntactic classes and the rules for combining them that vary from language to language. Only when a structure has been defined to be a Sentence by hierarchical classification of these rules does one consider whether the combination actual nouns, verbs and so on thus decreed combine in a meaningful way.
2. By contrast, *construction grammar* (Croft, 2008; Fillmore, 1976; Goldberg, 2003) is always focused on the challenge of how words are arranged *to convey (possibly novel) novel meanings.* One early motivation for this move from generative linguistics was that languages contain many idioms. Thus, to master the grammar of a language in meaningful way, one needs rules (often of much finer grain than those of generative linguistics) that can exploit the knowledge of such idioms in linking form and meaning. For example, a construction grammar offers the tools to distinguish the two forms of “he kicked the bucket” in “He kicked the bucket and then had to mop the floor” and “He kicked the bucket during Covid.” The general notion for BtMH is that early humans had no general syntactic categories such as Verb and Noun but did have a range of “protoconstructions” for linking, for example, (proto) words for certain types of action to those expressing objects with which or on which the action might be performed.

**FIGURE 1:**
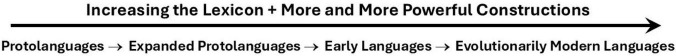
A gradient spanning from the earliest protolanguages to the verbal and grammatical richness of modern languages. There are no strict boundaries on the gradient.

Each (proto) language along the diverse paths to modern languages is *useful*—it helps people both to conform and to act more flexibly in a cultural milieu in which that language is shared. The transition from protolanguages (note the plural) to languages is a continuum (Figure 1). The earliest protolanguages had protowords that were close to being conventionalized pantomimes and so functioned at the bottom of the tower of abstraction. The gradient in Figure 1 proceeds as protowords yielded to words governed by constructions with lexicon and grammar growing to provide the ability to express a widening range of novel meanings.

With Wray (1998) 2000, BtMH insists that many protowords may have been *holophrases*—utterances that combined separate meanings even though there were originally no (proto) words to express these meanings. For example, a pantomime for opening a door has no separate pieces for “open” and “door.” BtMH explores how our ancestors *simultaneously* built protoconstructions and the protosigns they combine. It posits that the general ability for *complex action recognition* came (perhaps after tens of millennia) to support *fractionation* of protowords—breaking them into pieces that “were not there before” but came to be associated with their own meanings. The complementary process yielded constructions as a way to “put the pieces back together” so that, perhaps there would now be one-slot construction indicated by the word *open, open[X]*, and a new protoword for *door* so that the holophrase would be replaced by the structure *open[door]* —but now with the advantage that they could also combine pieces that had never been combined before. There is no claim in BtMH that this is the only way protowords form (see Arbib, 2012, p. 252–271).

Over time, BtMH suggests, constructions became merged or generalized, and complemented by new ones, and so the “slot fillers” went from having highly limited semantic variation to becoming abstract enough to form syntactic categories. As our physical and social worlds became more complex, aided in great part by the use of language, so did the lexicon and grammar of our languages have to grow. The process of *grammaticalization* (Kuteva and Heine, 2021) provided one tool whereby phrases that may have been strictly embodied became instead portions of grammatical constructions. Thus, in “I’m going to the store,” *going to* is strictly embodied but in “I’m going to go to the store after lunch” it is purely grammatical as a future marker. Humans have the capacity to recall the past, think about the future, and consider alternative, counter-factual events. This example of a future marker relates to a topic neglected in early work on MSH, the mental time travel (Suddendorf et al., 2009; Suddendorf and Corballis, 2007) that is essential to the modern capacity for narrative as another means for moving up the tower of abstraction. The power of language is that we can use it to convey new meanings and help catalyze the emergence of new concepts, though the full meaning of the concept may rest on experience beyond language (see §5). In particular, words and grammar work together to create *narratives* and to *define* new words that are immediately equipped with initial conceptual links but which will be enriched as they are later encountered in diverse contexts. Our discussion of *justice* gave a sketch of such processes at work.

## 4 Embodied simulation and metaphor processing

In this section we review an array of empirical evidence for cases where bodily knowledge appears to play a role in the construction of meaning across multiple levels of linguistic processing. In other, this section charts the roots of the gradient of abstraction and the tower of embodiment by shoeing how strictly embodied language may be transformed by metaphor to support usages that are no longer strictly embodied. We reiterate that the human ability to do this is supported primarily by changes in embrainment rather than in bodily form.

Embodied effects have been documented at the phonological level, as well as in semantic processing, both for language referring to actions and for certain abstract words and sentences, and even at the pragmatic level, affecting the interpretation of communicative intentions and contextual meaning. More recently, studies have shown embodied effects in the processing of negation, a logical operator at the boundary between semantic interpretation and syntactic structure. Our task in this section is to address these findings in a way that advances our account of the gradient of embodiment and tower of abstraction through the use of metaphor and grammaticalization. Consider the following examples

1. When we see someone grasping a hammer, we activate regions of the brain engaged in grasping a hammer, in some sense *simulating* the action;
2. When we hear a sentence like “Eduardo grasped the hammer” we may activate those regions so that in this sense our understanding of the sentence may involve *simulating* the action; and
3. A bodily metaphor like “He grasped the idea” may (but may not) invoke motor system activity for grasping so that it too involves bodily simulation.

These suggested a theory of *Embodied Simulation*^5^ motivated in part by findings on mirror systems but not limited to motor areas or the observation of goal-directed actions (Cuccio and Gallese, 2018; Gallese, 2008; Gallese and Sinigaglia, 2011). Embodied Simulation denotes the reactivation and redeployment of bodily knowledge for functions that extend beyond its original sensorimotor purposes.^6^ Gallese and Cuccio (2015) stress that “there are cases in which language… is not tied to any form of bodily knowledge … (e.g., when we talk about … moral judgment).” In this spirit, we demonstrate that Embodied Simulation contributes to generating lower levels of the tower of abstraction but may contribute to higher levels when abstract concepts are enriched by bodily metaphors. However, the necessary processing may involve brain regions far removed from the sensorimotor periphery that can, for example, support grammar use in humans who have developed in a language-rich milieu.^7^

### 4.1 Embodied metaphor processing

Embodied Simulation as a component of language has been particularly important in concert with *Conceptual Metaphor Theory* (Lakoff and Johnson, 1980), but not all metaphors are “embodied.” Metaphors are often viewed as providing mappings between distinct conceptual domains that allow understanding of the source domain to support thinking about the target domain (Black, 1962). In developing the theory of Embodied Simulation, metaphors are a cognitive mechanism where the source domain is provided by bodily *and other concrete* experiences (see also Gibbs, 2006; Kövecses, 2008). Such metaphors may enable the comprehension of abstract concepts. We consider bodily metaphors here as a particular instance of concrete concepts functioning within the source domain of metaphor.^8^ Gallese and Lakoff (2005) argued that data on the embodiment of metaphors show that Embodied Simulation provides the neural basis for Theory of Embodied Simulation.

Behavioral, neuroimaging and neurostimulation studies (Buccino et al., 2005; Glenberg and Kaschak, 2002; Glenberg et al., 2008; Hauk et al., 2004; Kemmerer et al., 2008; Sato et al., 2008; Tettamanti et al., 2005) have shown that processing bodily-related metaphors will often recruit related sensorimotor systems (for a discussion, Cuccio, 2022, 2025). For example, listening to “Mary grasped the idea” may activate hand-related areas of the motor cortex, suggesting (Cuccio, 2018) that recruitment of action and perception brain areas is an integral part of language comprehension. The theory of graded embodiment stresses that Embodied Simulation can be crucial in some meaning construction but can be unnecessary in others. Indeed, in some studies (e.g., Aziz-Zadeh et al., 2006; Cacciari et al., 2011; Raposo et al., 2009) the processing of bodily related figurative expressions did not activate the sensorimotor system, while some authors (e.g., Mahon and Caramazza, 2008) argue that activation of the simulation mechanism may be only a byproduct of language processing.

Frozen bodily metaphors seem to trigger less, or no, motor-system engagement. This suggests that frozen metaphors are interpreted at an abstract level without accessing the bodily experiences from which they originally drew their meaning. This relates to the notion that the tower of abstraction not only supports formation of abstract concepts but also supports processing that need not link these abstract levels with levels of concrete or bodily processing. The *origins* of such concepts may be embodied, but in their current usage their processing need no longer be embodied. Moreover, since many metaphors are based on non-bodily source domains, their origin as well as their processing may proceed at higher levels of the tower of abstraction. This does not deny that abstract concepts may enter into concrete discussions: a treatise on justice may have pages of text that do not link directly to bodily experience before linking to a specific case of, for example, grievous bodily harm. Moreover, some abstract concepts may be linked to embodied concepts. Physics, for example, relies heavily on metaphors having concrete concepts as source domain.

### 4.2 Different processing modalities in the comprehension of metaphors

Bowdle and Gentner’s (2005) *Career of Metaphor Hypothesis* holds that when a metaphor is still fresh, its comprehension takes place by means of *structural alignment*, a form of analogical thought that seeks out the relational patterns shared by the source and the target domain of the metaphor. Understanding, in this sense, is an active operation of mapping that also suppresses features that are not shared between the two domains. As the metaphor “freezes,” what was a process of applying a category from one domain into another (a Procrustean bed) is replaced simply by applying a new category that has developed within the target domain. Processing then may proceed entirely within the target domain as a new possibly abstract category but may occasionally exploit the added richness of invoking aspects of the earlier source. When there is no mapping back to bodily experience, the comprehension of such expressions becomes effectively disembodied. Consider the category “apple.” We can link the concept to numerous experiences, from cooking with it to eating it, each engaging different sensory and motor modalities. Yet the concept can also be invoked in a largely disembodied manner—when someone mentions having paid ϵ2.20 for a bag of apples, the term “apple” need not elicit any of those experiential associations. Similarly, the Deliberate Metaphor Theory (Steen, 2008, 2011) identified the “paradox” that most of the metaphors we use are not processed as metaphors. Lexical disambiguation refers to the process of selecting the appropriate sense of a word when several meanings are possible in a given context, they need not access the “literal” meaning from a bodily source domain. This absence is mirrored in the neural activation patterns associated with understanding such figurative expressions (Bambini et al., 2011). Analysis of these different processing modalities (i.e., analogical comparison and categorization) can offer valuable insights into the human capacity to ascend the “tower of abstraction.”

Furthermore, the distinction between analogical comparison, where a mapping between source and target domain takes place, and categorization, where no analogical mapping is at play, helps explain why simulation is not always engaged during metaphor comprehension.^9^

Taken together, the involvement of this bilateral network and the contribution of sensorimotor regions to metaphor comprehension indicate that linguistic interpretation extends beyond the traditional language areas. It draws as well on additional neural systems whose participation varies with the semantic and pragmatic properties of the input, and with the demands of grammatical parsing, where contextual and co-textual information, syntactic configuration, and part-of-speech cues shape the unfolding interpretation. The involvement of neural systems outside the classical language regions is accounted for within the framework set out by the gradient of embodiment proposal.

A recent study conducted by Cuccio’s research group examined the embodied nature of metaphor processing, focusing on how embodiment varies according to the degree of metaphor conventionality or novelty (Garello et al., 2024). They hypothesized that figurative expressions with different levels of conventionality engage the sensorimotor system to varying extents. To evaluate this hypothesis, they carried out a behavioral experiment comparing the processing of literal, idiomatic, conventional metaphoric, and newly coined metaphoric sentences.

In the experiment, participants judged the meaningfulness of each sentence by pressing a designated response key, allowing the experimenters to record both accuracy and reaction times. The data revealed that idiomatic expressions showed no distinction between the matching and mismatching conditions. By contrast, participants responded more quickly to literal and conventional metaphorical sentences when the preceding action was congruent with the sentence, relative to when it was incongruent. Interestingly, the opposite pattern emerged for novel metaphors: mismatching actions produced faster responses than matching ones, indicating an interference effect.

These outcomes suggest that the engagement of sensorimotor simulation differs according to the conventionality of the figurative expression. For literal sentences and conventional metaphors, observing a congruent action beforehand may prime motor representations that subsequently facilitate the processing of action-related language. The activation of pertinent motor features, in these cases, support the construction of meaning. Incongruent actions, however, may activate motor information that is irrelevant to the linguistic input, thereby interfering with the process of comprehension.

With novel metaphors, the interference observed in the matching condition may reflect a more deliberate search for meaning. Participants may retain aspects of the congruent action in working memory in an attempt to examine semantic correspondences between the action and the emerging metaphorical interpretation. Consequently, the processing of novel metaphors does not appear to show the rapid and automatic profile characteristic of literal and conventional metaphorical expressions.

For idioms, which were the most conventional stimuli in the set, the absence of differences between congruent and incongruent conditions aligns with previous work suggesting that idiomatic expressions no longer rely on the motor features originally associated with their constituent action verbs (Lauro et al., 2013; Yang and Shu, 2016). Because we directly access their figurative meanings, congruent actions do not enhance comprehension, nor do incongruent ones interfere with it. Processing, in this case, seems to not entail an analogical mapping. Categorization is the modality of processing underlying the comprehension of idiomatic sentences.

Overall, the findings support an embodied perspective on metaphor processing, while also indicating that the extent to which bodily knowledge is recruited depends strongly on how conventional or novel a given expression is. Similar considerations can be drawn in relation to the processing of metaphors in a second language. In this case, a metaphor that might be highly familiar and conventional for a native speaker might sounds less conventional and fresh for a second-language speaker. This is likely reflected in how the metaphor is processed and represented in the brain. A follow-up investigation (Garello et al., 2024) was carried out to assess this hypothesis and examine the embodied processing of metaphorical language in both first language (Italian—their native language, L1) and second language (English—their secondary language, L2). The participants were native speakers of Italian who had begun learning English after the age of ten. The experimental design mirrored that of the previous study by Garello et al.: participants assessed the meaningfulness of literal, idiomatic, and conventional metaphorical action sentences presented in both Italian and English. The results obtained in the L1 condition reproduced the pattern observed in the earlier study. Interestingly, in the L2 condition, a different profile emerged. Here, responses in all sentence types, including idiomatic expressions, were faster when the preceding action matched the sentence. One plausible explanation is that idioms often pose difficulties for L2 users, as idiomatic meaning tends to be culturally anchored. As a result, L2 speakers may be more inclined to interpret idioms compositionally, drawing on the bodily and motor representations linked to the action verb in order to derive meaning.

### 4.3 Embodied negation processing

We now address the embodiment of negation, the only logical operator whose processing has been examined from an embodied perspective. In doing so, we will examine both issues of embodiment and the role of grammar.

Although expressions such as “He grasped the idea” may have in part a strictly embodied origin in the act of grasping, but *idea* itself is not strictly embodied concept and resides at a high level of abstraction. The pronoun “he,” for example, plausibly derives from embodied social knowledge, recognizing individuals and perceiving gender as a socially salient, albeit abstract, category. Cross-linguistic evidence supports this view: Many Australian Aboriginal languages grammaticalize kinship terms, reflecting their centrality to social interaction (Evans, 2003). From a construction-grammar perspective, certain words do not carry independent semantic content but instead acquire meaning through the roles they play within grammatical constructions. Thus, “the” participates in constructions that map noun phrases to determiner phrases and signal discourse context. Use of “the idea” presupposes that the context has already identified a specific idea, whereas “an idea” signals the absence of such specification. Grammar thus provides structured resources for combining lexical items to generate novel meanings, which may subsequently be treated as embodied or disembodied wholes. Negation stands out as a unique case: it is a semantic concept that is syntactically instantiated through grammatical rules and structural positions, and that has been explored within embodied frameworks.

Recent research suggests that processing of linguistic negation *may* engage neural systems traditionally associated with the inhibition of motor responses (for a discussion, Beltrán et al., 2021; Montalti et al., 2024). Expressions such as “no” and “don’t” are among the most frequently heard words in early childhood and are commonly uttered by caregivers to prevent potentially harmful behaviors. As a result, linguistic negation is likely initially acquired within situations of action inhibition, and its earliest developmental stages are plausibly grounded in embodied experience (Cameron-Faulkner et al., 2007; de Carvalho et al., 2021; Hendry et al., 2022).

Beltrán et al. (2019) carried out an EEG study using the Go/NoGo task during the processing of sentences that referred to bodily actions and to mental actions and found that that negation consistently attenuated fronto-central theta activity associated with NoGo trials, regardless of whether the sentence content concerned physical or mental events [e.g., “Now you will (will not) wish a surprise”], whereas no differences emerged between affirmative and negative conditions in Go trials. In addition, a parallel interaction was observed in the beta frequency band over right-frontal regions. These results suggest that the overlap between negation processing and inhibitory control is not confined to action-related language, but generalizes across distinct semantic domains.

Negation’s general importance as a logical operator is that the motor correlate is not a neural activity that encodes an action that embodies part of a verbs meaning (as when motor activity for grasping is evoked even for “he grasps the idea”) but that negation provides a “neural activity” that reduces the motor activity for the relevant action, even if not inhibiting it completely. Moreover, as linguistic competence expands, and in line with the gradient of embodiment proposal, speakers become capable of processing negation in increasingly abstract and disembodied contexts, such as in the mathematical statement “the number of prime numbers is not finite,” where no sensorimotor mirroring effects should be expected, unlike the above example of mental action. However, since the involvement of resources for motor response inhibition has never been empirically investigated in these highly abstract contexts, this remains an open issue.

### 4.4 Graded embodiment in language processing

The previous sections suggest two important considerations. The first is that a more-fine grained notion of metaphors and their processing modalities is fundamental. We need it to understand the role of the body in the process of construction of figurative meaning. The contribution of the body can be understood as spanning a continuum from strictly embodied to largely disembodied processing. This gradient cuts across the entire tower of abstraction. At the lower levels of the tower, we find concrete concepts that are closely tied to sensorimotor experience. Higher on the tower, however, both possibilities remain open: some concepts are still embodied, particularly those structured through bodily-based metaphors, while others are disembodied. The latter can arise from multiple sources, including metaphors whose original analogical links have faded, and culturally shaped constructs. In other words, embodiment is not a binary concept. We cannot just say that a meaning is or is not embodied. Embodiment is a gradient. Alongside bodily grounded knowledge, purely symbolic representations encoded in the classical language areas are invariably required.

## 5 Neurolinguistics, LLMs, and (dis)embodiment

It has been claimed that Large Language Models (LLMs) are models of the language system of the brain, and that this discredits the claim that formal language competence is embodied (Fedorenko et al., 2024a; Mahowald et al., 2024). We first set the stage by noting how neurologists discriminate between apraxia and aphasia. We then examine, briefly, the impressive performance of LLMs. They show how much of language can be dissociated from strong embodiment. Nonetheless, we argue, contra Fedorenko et al., that this does not discredit our claim that *how humans use language* can best be understood within our framework of graded embodiment.

### 5.1 Apraxia and aphasia

We noted at the end of §3.1 that the biocultural evolution that distinguishes the embrainment of modern humans from that of LCA-ga developed brain regions distinct from “basic” sensorimotor systems that supported language. To reinforce this point, we summarize how classic neurology revealed the dissociation between language processing and action processing.

Apraxia (from the Greek *a+praxis, without action*) is difficulty with motor planning to perform tasks or movements caused by damage to the brain. Crucially, the problem is neither one of perceiving affordances in the environment nor of the ability to control the muscles. It is specifically the ability to appropriately schedule actions to achieve one’s goals. The varieties of apraxia include *apraxia of speech*, having difficulty planning and coordinating the movements necessary for speech (e.g., tomato → motato); *ideational/ conceptual apraxia*, where patients may have impaired ability to select and execute an appropriate motor program (given a screwdriver, a patient may try to use it like a pen to write with, or may complete the actions of a program in the wrong order, such as buttering bread before toasting it); and *ideomotor apraxia*, having deficits in the ability to plan or recall motor programs (a patient can explain how to perform an action, but be unable to pretend to comb their hair when instructed—and yet may be able to comb their hair when provided with the affordances of an actual comb). Different areas of brain damage may cause different forms of apraxia. Lesions may impair sequencing of vocal actions with little damage to sequencing of practical actions and vice versa.

Aphasia (from the Greek *a+phásis, without speech*) degrades a person’s ability to comprehend or formulate language. As for apraxia, there are diverse forms of aphasia and no complete correlation of type of dysfunction with damaged brain regions. For example, aphasias may impair production or comprehension differentially, and aphasia in bilinguals (or people fluent in both a spoken and a signed language) may impair use of one language more than in the other. However, a person with aphasia may still have use of formulaic language—hitting his thumb with a hammer, a patient may still swear proficiently. Crucially, aphasia is distinct from impairment of visual or auditory perception of words or the mechanics of producing them. Instead it involves impairment of the individual’s language cognition, a complex that integrates the socially shared lexicon and grammar of their language with an understanding of how to employ language in different contexts.

The brain is a highly distributed system, but different regions seem more crucial to some functions than others. Crucially, normal function may involve cooperation and competition between diverse areas of the brain as well as between different individuals. For example, when we say to someone “Get the pen,” success of the request requires not only language comprehension and the wish of that person to satisfy the request but also their ability to use that comprehension to first visually locate that pen and then to coordinate vision and action to retrieve it. However, if we have explained an idea to someone, and they look puzzled, you might ask “Don’t you get it?,” the task of comprehension is more abstract and engages neither vision nor manual action.

Apraxia of speech can be dissociated from apraxias for practical action and from aphasia, though some patients may exhibit multiple syndromes. However, there is no neat correlation of the anatomy of damage with the type of impairment—stroke and head trauma are no respecters of the neuroanatomist’s parcellation of brain regions. Moreover, the brain’s competition and cooperation of multiple subsystems implies that brain lesions may impair function not only by removing relevant circuitry but also by impairing pathways necessary to the integrated performance of several regions. Conversely (recall our mention in §3.2 of neural reuse and cortical recycling), since the brain is a highly adaptive system, some damage to the brain can be compensated for as the remaining systems learn to recover much of the impaired functionality.

Given all this, to a first approximation language processing in the brain can be dissociated from the sensorimotor mechanisms for practical actions. But since language use, practical action, and social coordination are so intertwined, there must be strong coupling between brain systems for praxis and language. How does this relate to our account of graded embodiment of language and the tower of abstraction? As Cuccio and Gallese (2018) observe, speakers undeniably possess a form of linguistic competence that can be described independently of bodily experience, structured around lexical items and morphosyntactic rules. But to what extent can such competence be identified with meaning itself? Pragmatically, meaning does not reside in static representations but is constituted in and through acts of use. Viewing the mental lexicon as containing delimited definitions, as in a dictionary, proves insufficient. Lexical items and morphosyntactic rules operate as prompts to activate a broader background knowledge Evans (2006) characterized as encyclopedic knowledge (and see Blondin Masse et al., 2013). Our knowledge draws on perceptual, bodily, emotional, and culturally embedded forms of experience alongside linguistic information. Meaning, therefore, cannot be assumed to be uniformly disembodied or embodied; instead, in line with the Gradient of Embodiment proposal, it emerges through varying configurations of linguistic and non-linguistic resources, depending on the circumstances of use.

### 5.2 LLMs do not discredit claims for graded embodiment^10^

LLMs have apparently mastered natural language without bodies whereas humans have eyes, ears, hearts, guts, arms, legs, and so on. LLMs are trained through extensive exposure to vast collections of human-produced texts. This immersion in patterns of use, rather than access to abstract lexical definitions or grammatical rules, enables them to generate responses that resemble human language use. This provides a compelling illustration of the claim that meaning does not arise from static linguistic knowledge alone, but is grounded in use, emerging from contexts and practices reflected in actual linguistic interaction.

Does this support a claim for linguistic competence without grounding in bodily experience? To assess this, we need to contrast different notions of *linguistic competence*. For generative linguistics it referred to the syntax alone (form without meaning), offering a set of rules for building syntactic structures (Radford, 2004). Construction grammar was one countervailing approach in which form and meaning were intertwined (Fillmore, 1976). Each of these seems compatible with a purely symbolic approach. But other mechanisms must come into play if one wishes to understand human language use emerges through the experience of each individual child (MacWhinney et al., 2022).

The lack of embodied experience is in some ways a strength of LLM’s in that they can be trained on vast bodies of data much more quickly than learning from actual interaction with the external world, but they cannot use language in ways that are directly informed by such experience rather than through statistical relations with other sentences that have encoded parts of the experience relevant to the current processing of texts.

The trained behavior of an LLM shows that these words are treated as forming syntactic, semantic, and even pragmatic patterns even though there was no explicit training in these categories or direct experience of the world. But in what sense does an LLM *understand* “kick the ball”? It has no way, for example, to inspect a video and answer the question “Is anyone in this video kicking a ball?” An LLM as such cannot do this, unless it is coupled to a visual AI system with a sophisticated language interface. Implementations of such systems now exist, but they weaken any claim that a system uncoupled to a perceptual system can fully understand language. A robot further requires a motor system engaged in understanding “kick the ball” in a manner distinct from understanding “the bird flies over the tree.” It is only through coupling with sensory and motor interfaces that will enable LLM to *begin* to “understand” how language relates to the external world. On the other hand, sophisticated information that has already been encoded in written text can indeed be deployed impressively by an LLM. In contrast to BtMH’ s hypothesis that protolanguage first evolved to coordinate behavior and share attention to salient features of the environment, AI develops systems “symbols first”—reversing the order of human biocultural evolution. It starts with a language system that, through immense training, can respond to language input with language output that is often useful to humans who supplied the additional prompt. But only the development of AI systems for perception and for control of a “body” can link certain of an LLMs utterances to actual states of that “body” in the world.

### 5.3 Do LLMs model the brain’s language system?

There is a rapidly growing literature comparing LLMs with language systems in the human brain. Ev Fedorenko’s group has been especially productive in this area, and so their work will be the focus of this brief section in which we argue (briefly in the present paper) as we argue that the language-processing mechanisms of the human brain are very different from those of the human. Nonetheless, we can agree that although much of human language processing is strictly embodied so too can much of it proceed in a fashion independent of strict embodiment.

Schrimpf et al. (2021) and Tuckute et al. (2024) have shown that activations in LLMs correlate with coarse measures of neural activity in the brain’s language network during tasks like sentence comprehension and assert that this implies that the brain’s language system operates in a modular, abstract fashion, rather than being tightly coupled to sensorimotor systems. However, these correlations are indeed coarse and do not establish that the circuitry of brain regions is like that of transformers. Indeed, their feedforward structure differs from the circuitry of human neocortex, and the ability to remember the exact words of a lengthy prompt plus the response so far is beyond the memory capacity of most humans. In particular, internal activation of an LLM (as distinct from the strength of connections) is recomputed at each iteration, lacking the diverse memory structures involved in the human brain (Squire and Wixted, 2011).

Citing fMRI-based neurolinguistic studies, Fedorenko et al. (2024b) argue that language evolved primarily for communication rather than for internal thought. They view language as a modular cognitive system, specialized for encoding and decoding structured sequences of symbols, rather than being a scaffold for embodied simulation or sensorimotor grounding. This seems like a false dichotomy as evidenced by our work on both BtMH (§3) and embodied simulation (§4). We argue, then, that the human “language system” cannot be seen as a separate module linked to prompts and responses by pre-learned embeddings. Rather, it develops as the child acquires language while interacting with other people and the physical environment. The rich interconnections of circuits in and across human brain regions imply that learning cannot be neatly confined to one part rather than another. Our graded approach to embodiment explores how the human language system can be directly coupled to sensorimotor planning or simulation in some cases but that much processing could be done in the brain occurs at levels higher up the tower of abstraction. This leaves open the question of to what extent the structures that make abstractions possible are to be thought of as falling within other systems strongly coupled to the language system versus the language system itself.

## 6 Discussion and future directions

The basic plan of the human body is in many ways similar to that of great apes, but apes do not have language. We thus emphasized *embrainment* in suggesting that humans have language because their brains evolved to be able to support manual imitation and pantomime related to embodied behavior, and that these in turn supported processes of conventionalization that led at first to simple forms of protolanguage. These communicative capabilities in turn supported many millennia of cultural evolution that enabled humans to develop languages with rich lexicons and construction grammars as well as social and physical environments in which children could acquire the language of a community and, for some, contribute to the ongoing process of cultural change. The ability to create novel meanings by the use of grammar, to use tools like negation to describe entities that did not exist and yet could help explain experience, and to define new words all contributed to the extension of language via metaphor and processes of abstraction to remarkably expand human cognition by moving along the gradient of embodiment and/or up the tower of abstraction.

We noted that “language is embodied” has two distinct meanings. We turned from the overly general “language use depends on brain, body and culture” to introduce a notion of “strict embodiment” that directly engages certain elements of language with the sensory and motor systems of humans engaged in practical bodily interaction (as distinct from communicative action). Our *gradient of embodiment* proposal then explores how language use can range from strictly embodied (“he grasped the hammer”) to fully abstract (“philosophy,” “justice”). We claim that strictly embodied knowledge provides the phylogenetic and ontogenetic grounding of linguistic meaning, but that much adult language use resides “high” in a *tower of abstraction.*

We showed how Embodied Simulation provides one of the mechanisms for meaning-making within a pragmatic, usage-based account of language. The meanings expressed by words are shaped by the situations in which they are used. For example, the word *milk* may evoke the experience of drinking milk but uttering the word may function as a request to add an item to a shopping list, as an instruction to retrieve milk from the refrigerator, and so on—processing of such words may but need not trigger the mechanism of simulation, depending on the context of utterance.

Egorova et al. (2016), had participants view a series of videos in which identical one-word utterances occurred within distinct communicative situations, either serving to label objects or to solicit them from an interlocutor. Participants watched short videos displaying two persons (a “Speaker” and a “Partner”) seated at a table with 12 objects placed before them. In each trial, the Partner first produced a contextual sentence that established the pragmatic frame for the ensuing interaction. This sentence was followed by a one-word utterance naming an object on the table, spoken by the Speaker. In each case, visual attention and object recognition must have been involved. But when the utterances were understood as requests, participants’ neural responses were observed in bilateral premotor regions, as well as in the left inferior frontal and temporo-parietal cortices, apparently simulating the action representation of the Partner. Presumably, the left angular gyrus, a region implicated in integrating lexical form with referential meaning, must have been active in both cases, but predominated when interpreting the utterances as names elicited in the label case. In the authors’ view, these results indicate that the comprehension of utterances as distinct types of communicative acts is associated with different but possibly overlapping patterns of brain activation, supporting the view that different speech acts rely on partially distinct neural mechanisms. Such studies suggest the interest of new studies of embodiment in relation to the varieties of apraxia and aphasia, perhaps with special attention to cases where patients exhibit symptoms that overlap both.

Such empirical findings clarify the notion that words such as *milk* are not restricted to embodied meanings. Rather, they illustrate a general feature of linguistic practice: words, whether concrete or abstract, do not carry meanings fixed independent of context. Meanings are context-sensitive and dynamically constructed in use. This variability is not evidence for a disembodied conception of language, but a consequence of how language functions as a tool for action and coordination within situated practices.

We have offered construction grammar, rather than generative linguistics with its divorce of syntax from semantics, for our usage-based approach to the gradient of embodiment and abstraction. In BtMH’s phylogenetic account of language evolution, grammar was proposed to have emerged in part from the fractionation of the holophrastic structures characteristic of protolanguages (§3.2). Through processes of cultural transmission, individual constructions were gradually combined, extended, and supplemented, such that the elements occupying slots in constructions moved from exhibiting narrowly constrained semantic variation to displaying a level of abstraction sufficient to support the emergence of syntactic categories. On this view, the lexicon and grammar did not develop independently but coevolved as part of a single cultural dynamic, ultimately yielding fully articulated linguistic systems, in which form and meaning were integrated. In present-day language use, grammar therefore occupies a distinctive position within the framework of a gradient often combining embodiment and abstraction: it is neither elevated to the status of the primary explanatory core of language nor straightforwardly reducible to embodied mechanisms, but rather marks the domain in which the embodied foundations of linguistic meaning have become least transparent.

Negation remains, to date, the only phenomenon for which empirical evidence bears directly on embodied involvement at the grammatical level (§4.3). We have suggested that its original role as a modulator of embodied action evolved, through grammaticalization (like *going to* becoming a future marker), to make possible the evolution of constructions that apply negation to even the most abstract of statements. Crucially, different languages offer different embodied bases for the emergence of words and constructions that play variations on similar semantic roles. This is demonstrated by Heine’s (1997) treatment of the diverse grammaticalizations that lead to variants of *have* in different languages. This suggests that it would be fruitful to explore the grammaticalization literature to find terms and constructions that do and do not carry “echoes of embodiment.”

In §5, we briefly distinguished the notions of apraxia and aphasia to note that human brain regions supporting language and action can be to some extent dissociated. We then noted that LLMs can respond to lengthy queries or requests written in some human language and respond at great length in that same language. There is a sense in which we might say that the computer on which the LLM is implemented has mastered some important aspects of language use, and it would be hard to claim that such use is embodied in a way that resembles the human body. Nonetheless, we were able to conclude that (a) LLMs do not constitute a model of language-processing areas of the human brain, (b) that in some ways LLMs do not understand the meanings of words they use and sentences they generate, and (c) the challenges of coupling LLMs to, e.g., visual systems and robot control systems, hints at ways portions of AI research may be structured explicitly to help us better understanding how language is enriched by bodily experience.

In much human language use, embodiment and abstraction are intertwined, and we have seen how bodily metaphors may enrich discourse employing even abstract terms like *bureaucracy.* Indeed, while the continuing cultural evolution of language creates new words and forms high up the tower of abstraction to support rather abstract discussions, their utility often resides in the ability to link these abstractions to embodied discourse. For example, debating about the fairness of a particular court case may be enriched by deploying the notion of *justice* while enriching it in turn.

Given the above hints, we predict that our framework will support new lines of research recasting the debate over the embodiment of language and cognition. The notions of embrainment, graded embodiment, and the tower of abstraction allow research to move beyond the familiar opposition between embodied and disembodied conceptions. Rather than asking whether language is embodied *simpliciter*, we argue that different linguistic processes and representational levels stand in different relations to bodily experiences. While early stages of language evolution and acquisition are deeply shaped by embodied interaction, successive layers of abstraction that are shaped by cultural evolution and grammaticalization progressively loosen this connection but do not sever it entirely.

## Data Availability

The original contributions presented in the study are included in the article. Further inquiries can be directed to the authors.

## Footnotes

1 Arbib (2012) offers a book-length exposition, but MSH has been critiqued and updated over the years. For varied contributions to developing a new road map for investigating how the brain got language (see Arbib, 2020).

2 The form “proto-X” indicates something with some, but by no means all, the attributes of X that nonetheless provided an evolutionary precursor of what X provides for modern humans.

3 See further Slocombe et al. (2011) and Fröhlich et al. (2019). Arnon et al. (2025) focus only on vocal learning and speech production in the evolution of language.

4 Chapter 2 of Arbib (2012) presents the key ideas in a more leisurely fashion, providing extensive references, and relating these ideas more fully to BtMH.

5 de Bruin and Gallagher (2012) are among those who have criticized this concept of Embodied Simulation, while Arbib (2008) stresses that this is a long way from the engineer’s sense of simulation which would involve, e.g., computing the muscle commands needed to move a specific hand and fingers to grasp a specific object.

6 Unless noted explicitly to the contrary, our notions of embodiment exclude here peripheral systems engaged in receiving and producing the sounds (or other meaningless units) of language.

7 Greenfield (1991) found that children parallel their capacity to master hierarchical complexity both in the domain of language and goal-related action. Noting this, Gallese (2008) hypothesized that Phrase Structure Grammar is the output of a cortical premotor network originally evolved to control/represent the hierarchical structure of goal-related action. §4.3 exchanges Phrase Structure Grammar for the integration of form and meaning of Construction Grammar, and sees the necessary processing as falling outside the premotor areas that contain mirror systems.

8 It is worth noting that, although the concrete-abstract metaphorical mapping might be the most discussed and the ones that allows the grounding of abstract concepts onto bodily experiences, still examples of concrete-concrete, abstract-concrete or abstract-abstract mappings are also discussed within Theory of Embodied Simulation. However, within the Theory of Embodied Simulation, concrete-abstract metaphors are certainly the central case.

9 Neuroimaging studies have suggested that the processing of figurative meaning recruits a bilaterally distributed network (see Cuccio, 2022 for a discussion). This constellation of regions typically involves the inferior frontal gyrus, both left and right (BA 45) and, on the left, BA 47, together with the temporal–parietal junction in both hemispheres (BAs 22 and 39). Complementing this fronto-temporo-parietal circuitry, activity also emerges in the right anterior cingulate cortex (BAs 24 and 32), as well as in the right dorsolateral prefrontal cortex, particularly the middle frontal gyrus (BA 9) (Hagoort and Levinson, 2014).

10 Supplementary Appendix presents a brief introduction to LLMs that corrects misconceptions about them that distort some of the debate over their capabilities.
