# Supplementary material for: The body in language, the language beyond the body: embrainment and graded embodiment in the evolution and use of language
Source: Front Psychol. 2026 Apr 10;17:1774505. doi: 10.3389/fpsyg.2026.1774505 (PMC13107645; doi:10.3389/fpsyg.2026.1774505)
Supplement: Supplementary file 1 [file Supplementary_file_1.docx]

# Appendix: Introducing the Structure of LLMs

**Supplementary Material for** **“The Body in Language, the Language Beyond the Body: 
Embrainment and Graded Embodiment in the Evolution and Use of Language” by Michael A. Arbib and Valentina Cuccio.**

Starting in 2022, there has been remarkable success in training vast computer-simulated networks of artificial adaptive neurons (based very loosely on a few properties of biological neurons) to generate Large Language Models (LLMs) that are able to respond to a prompt that may run to several paragraphs in natural language with responses, again in natural language, that appear informative and often prove useful to the person who posed the prompt.

LLMs are trained on massive corpora of written language. Training is based on predicting the next word to follow an input sequence of words. However, once they are trained, LLMs go far beyond simply “predicting the next word”, generating and updating an internal state that corresponds to a longer-term plan, as in the case of responding to a prompt to compose a limerick where the last word of a line may be decided before the preceding words of the line are generated.

As a basis for the discussion of LLMs in §§5.2 & 5.3, we offer a high-level view of how they process prompts to provide responses. More generally, the structure explained here is called a *transformer* – it becomes an LLM when the overall input (the *prompt*) and the response are sequences of words.

*Figure 2: An Input-Output View of an LLM after the first j words of the response have been generated.*

[Alt text: An input prompt and output sequence are passed up through a sequence of layers to generate the next output. This is fed back so the process can repeat.]

The overall structure of an LLM (Figure 2) is designed to respond to an input “prompt”, a sequence of words, to produce a response as another sequence of words. The system combines a feedforward network with a feedback loop that carries one word at a time. In each iteration, the input to the feedforward network is (i) the full prompt as well as (ii) the words generated so far for the response, while its output is (iii) its decision on the next word of the response. This word is then added to the component (ii) of the LLM input, and the feedforward process is repeated. This return of the latest response word is the only feedback path in the network. These iterations continue until the full response to the prompt is read off from (ii).

The input and output sequences of LLMs are word *embeddings*, not sequences of words themselves. Instead, each word is broken into one or more tokens, and each token is embedded as a vector of even hundreds of dimensions. The embeddings also encode the position of tokens in the prompt. Components of the vector represent different aspects of each token with a distributed representation learned based on the notion that words that are used in somewhat similar contexts will be represented by vectors that at least in some dimensions are very similar, capturing some aspects of their shared meaning. The embedding is used to encode words for the input to the LLM, while at the LLM’s output requires a pseudo-inverse to extract words that match the embedding*.*

An LLM generates series of output strings, but many of those strings do not look “acceptable” to humans. The main reason LLMs got as powerful as they did was because of the addition of a complementary process called reinforcement learning to assess a subset of the prompt-response pairs with positive reinforcement based on responses that humans judge more acceptable.

*Figure 2:3 Overall anatomy of an intermediate layer of the LLM.*

[Alt text: All layers of an LLM receives an embedding of the input prompt. Layer k also receives an embedding of the LLM’s next output sequence as estimated by Level ki1 and produces an estimate of the embedded output sequence to relay to level k+1. The text notes exceptions for the first and last layers.]

Figure 3 shows the typical structure of the layers of the LLM. Before training the structure of the N layers will be the same, but after training the internal weights will differ between copies in different layers. The input to each layer comprises (a) the embedding of the input prompt and (b) the previous layer’s estimate of the output sequence. (b) is different for each layer. However,

- For layer 1, input (b) is the embedding based on the latest version of the response generated so far.
- The level N estimate of the embedded output sequence is decoded to extract a choice for the next output word.

This specialization of layers 1 and N is not made explicit in the founding paper on transformers (Vaswani et al., 2017) which defines the internal structure of encoders and decoders in terms of a collection of adaptive feedforward neural networks that include what they call “attention units”.

To reiterate, a key point of this Appendix is that, once they are trained, LLMs are not “predicting the next word” but rather generating and updating an internal state that corresponds to a longer-term plan, but then producing a word as output before updating the plan to ground the next word.
